# Coping and Anxiety in Caregivers of Dependent Older Adult Relatives

**DOI:** 10.3390/ijerph16091651

**Published:** 2019-05-12

**Authors:** Margarita Pérez-Cruz, Laura Parra-Anguita, Catalina López-Martínez, Sara Moreno-Cámara, Rafael del-Pino-Casado

**Affiliations:** 1 University Hospital “Dr. Sagaz”, Jaén, 23071 Jaén, Spain; mperezc23@gmail.com; 2 Department of Nursing, School of Health Sciences, University of Jaén, 23071 Jaén, Spain; cmartine@ujaen.es (C.L.-M.); smcamara@ujaen.es (S.M.-C.); rdelpino@ujaen.es (R.d.-P.-C.)

**Keywords:** caregivers, older adult, coping, burden, anxiety

## Abstract

The aim of this study was to analyze the relationship between coping and anxiety in caregivers of dependent older adult relatives. A cross-sectional study was carried out in the province of Jaén (Andalusia, Spain). The convenience sample consisted of 198 family caregivers of dependent older adults. The main measurements were anxiety (Hamilton scale), coping (Brief COPE), subjective burden (Caregiver Strain Index), objective burden and sex of the caregiver. The main analyses were bivariate analysis using the Pearson correlation coefficient, and multivariate analysis using multiple linear regression. An independent regression model was developed for anxiety and each type of coping, adjusting for sex, subjective burden and objective burden. Our results demonstrate that anxiety was negatively associated with planning (partial r = −0.18), acceptance (partial r = −0.22) and humor (partial r = −0.20), and it was positively associated with self-distraction (partial r = 0.19), venting (partial r = 0.22), denial (partial r = 0.27) and self-blame (partial r = 0.25). Planning, acceptance and humor coping strategies may be protective factors of anxiety. Strategies such self-management, relief, denial and self-blame may be risk factors for anxiety.

## 1. Introduction

The aging of the population that has occurred over recent decades has led to a considerable increase in situations of dependence in older persons and demand for long duration care. In Spain, this care is mainly carried out by relatives, as well as in other countries with profiles distinct from ours [1]. More specifically, it is the wife who carries the largest part of the care burden [2] and becomes the main personal caregiver. The main personal caregiver is considered as the person that takes on the permanent care of a relative without receiving any economic remuneration for it [3].

Those that take on the care of a dependent person find their lives radically affected, as normally they need to combine this with other work and family responsibilities [4]. Moreover, studies [5] indicate that the perceived needs of these caregivers are complex and always evolving. All the above place them in a situation of great vulnerability to the stress that caring entails, with an important impact on their health [6].

Existing evidence shows conflicting results on whether caring for a dependent older person impacts negatively on the physical and emotional health of the caregiver [7,8]. The negative emotional consequences are the most important, with anxiety being one of those with the greatest impact and transcendence [9,10].

The prevalence of anxiety varies depending on the type of person cared for, varying between 21.4% in caregivers of relatives who suffer a stroke [9], and 43.6% in caregivers of relatives with dementia [10].

The appearance of anxiety in caregivers is related to the sustained stress that caregiving entails. Distinct models and theories have tried to explain the stress as a consequence of caring for a relative. The majority of these are based on the transactional theory of stress by Lazarus and Folkman [11]. Among these, the most used is the multi-dimensional model of stressor agents by Pearlin et al. [12]. In this model, the appearance and proliferation of stress is related to five factors: the antecedents and context of care; objective primary stressors, or objective burden; subjective primary stressors, or subjective burden; secondary stressors; and mediators or moderators of the impact of caregiving, such as social support or coping mechanisms [11]. Objective burden consists of care demand and the intensity of attention given. Subjective burden is the caregiver perception of the care demands as stressors. Secondary stressors comprise role strains and intrapsychic strains.

In this context, coping is defined as “those constantly changing cognitive and behavioral efforts that are developed to manage the specific external and/or internal demands that are perceived as exceeding or overpowering the resources of the individual” [13]. Coping is classified, depending on the orientation of the efforts, as problem-centered coping (directed toward resolving the problems) and emotion-centered coping (focused on managing emotions), or, depending on the nature of the aforementioned efforts, as active coping/approximation (aimed at resolving, minimizing or re-evaluating the problem) or passive coping/avoidance (avoidance of the problem) [14].

Studies that analyze the relationship between coping and anxiety in caregivers of dependent older persons are scarce [15], although sufficient to establish differential relationships between the types of coping and anxiety. Few studies have shown that passive coping strategies, such as denial or evasion, are associated with higher levels of anxiety in caregivers [16,17]. The results in studies for the use of active coping strategies, both aimed at resolving the problem and managing emotions, are heterogeneous. In terms of a problem-centered coping strategy, some studies do not find any association with anxiety [16,18], while others show that this type of coping is accompanied by greater anxiety [19]. Literature shows similar findings for emotion-centered coping, with some studies that show that it may have a negative effect on anxiety [20], and others that show a possible beneficial effect [16].

The clarification of the role that different coping strategies play in the terms of anxiety could provide significant knowledge that assists in the prevention of this anxiety, improving the quality of life of caregivers of dependent older adult relatives.

The present study aims to describe the most frequently used coping strategies by caregivers of dependent older adult relatives, and to analyze the relationship between coping and anxiety in these caregivers, controlling for possible confounding factors.

## 2. Material and Methods 

### 2.1. Design

This research was conducted using a cross-sectional design.

### 2.2. Participants

The main caregivers of dependent older adult relatives in the Primary Care District of North Jaen, Spain. A convenience sample was selected among caregivers of older persons who were seen in a medium-stay care center from July to September 2015. The inclusion criteria were: (1) primary caregivers (those who taking the responsibility for care and delivering the largest amount of care), (2) not receiving any economic remuneration, (3) for a relative older than 65 years, (4) who was dependent in at least one of the basic activities of daily living (ADLs). Participation in the study was offered to 213 caregivers that met the inclusion criteria, of which 198 agreed to participate and 15 refused to participate in the research.

According to Cohen [21], a sample size of 198 provides a minimum power of 82% to detect a r^2^ of at least 3.5% attributable to one independent variable, in a linear regression that has been adjusted for four additional variables with a r^2^ of at least 14%, with a statistical significance level of 5% (calculations performed with PASS 11 software (NCSS, LLC. Kaysville, UT, USA)). 

### 2.3. Measures

Dependent variable: the version of the Hamilton Anxiety Rating Scale validated in Spain [22]. This scale has 14 items. The interviewer scores each item from 0 to 4. Its score ranges between 0 and 56. A cut-off point of 14 has been proposed to detect clinically manifest anxiety [23]. It was validated in Spain by Lobo et al. [22] with good psychometric properties including internal consistency (Cronbach’s alpha of 0.89), test–retest reliability and inter-observer reliability (intraclass correlation coefficients of 0.98 and 0.92, respectively).

Independent variable: caregiver coping, measured with the abbreviated Spanish version of the Carver COPE questionnaire (Brief-COPE Inventory) validated by Crespo and Cruzado [24]. This questionnaire contains 28 items, and each item has a graduated scale from 1 (never) to 4 (always). The items are classified into 14 subscales (two items per subscale, with a score ranging from 2 to 8): active coping (initiate direct action, increase efforts to eliminate or reduce stress), planning (thinking about how to cope with stress, planning action strategies), positive reframing (looking for the positive side of the problem), acceptance (accept that the problem is real), humor (make jokes about stress or laugh at the stressful situation), religion (increase participation in religious activities), use of emotional support (affection and understanding), use of instrumental support (receiving help for tasks), self-distraction (engage in distraction activities such as reading, going to the movies, etc.), denial (deny the reality of the stressful event), venting (expressing or releasing feelings of emotional stress), substance use (use of substances such as alcohol and drugs to feel better), behavioral disengagement (neglecting responsibilities and abandoning stressful tasks) and self-blame (criticize and blame themselves for what happened). The internal consistency ranges from 0.52 (substance use) to 0.90 (religion).

Potential confounding variables: subjective burden, objective burden (functional capacity, cognitive decline, daily hours of basic ADL assistance and number of basic ADLs assisted with) and sex of the caregiver.

Caregiver subjective burden was measured using the Spanish version of Robinson’s Caregiver Strain Index by López Alonso and Moral Serrano [25]. This index is a 13-question tool with dichotomous responses (yes/no), with a score ranging from 0 to 13. This index has good psychometric properties (Cronbach’s alpha of 0.86) and construct validity [25]. Functional capacity was measured using the version of the Barthel Index validated in Spain by Baztán et al. [26], which is a 10-item scale with a theoretical range from 0 to 100. The Spanish validated version has adequate psychometric properties (weighted kappa coefficient for intra-observer of 0.98 and inter-observer of 0.88). Cognitive decline was evaluated with the Spanish version of the Pfeiffer test by Martínez de la Iglesia et al. [27], which included 10 dichotomous questions with a theoretical score range of 0–10. This version has been validated in Spain with a sensitivity of 85.7% and a specificity of 97.3%.

### 2.4. Data Collection

Data were collected from July to September 2015 by highly qualified nurses with a least five years of experience in the care of caregivers of dependent older persons. These nurses received a five-hour training session to guarantee the quality and uniformity of the data collected. This training session included recommendations on conducting interviews, the use of the measuring tools used in the study and data encoding.

### 2.5. Ethical Considerations

To guarantee confidentiality of all data collected in this project, all information was registered in an anonymous manner, strictly following the data protection laws and regulations in Spain. 

In addition, informed consent was obtained from all participants in the study. Ethical approval for this study was obtained from the Institutional Review Board (0903201201).

### 2.6. Statistical Analysis

For the descriptive analysis, means and standard deviations were calculated, as well as the corresponding 95% confidence intervals (CIs) for the proportions and means. The Pearson correlation coefficient (r) was used for the bivariate analyses, unless normality assumptions were not met, in which case the Spearman rank correlation coefficient was used. Multiple linear regression was used for the multivariate analysis, with prior confirmation that the linear regression assumptions were met: normality (Kolmogorov–Smirnov, normal P-P plots); linearity (partial regression graphs); homoscedasticity (standardized residual dispersion plots and standardized predictive); independence of errors (Durbin–Watson statistic); and non-collinearity (collinearity diagnostics). We performed independent regressions for each coping dimension (plus control variables) because collinearity was high when all coping dimensions were included in the same regression model (tolerance was <0.35 for four coping dimensions, and 0.36–0.6 for other four coping dimensions). Statistical significance was set at 0.05. All analyses were performed using the statistical package SPSS v. 19.0 (IBM Corp, Armonk, NY, USA) for Windows, except for the calculations of the CI for proportions, for which EpiDat (version 3.1, Pan American Health Organization, Washington, DC, USA) was used. 

## 3. Results

The profile of the caregiver was a woman (89.4%) or daughter or son (57.1%) of the person cared for, with an average age of 58 years. The profile of the dependent older person receiving care was characterized as a 78-year-old male with cancer (24.7%). The descriptive data of the study sample are presented in Table 1.

The mean anxiety of the caregivers, measured with the Hamilton questionnaire, was 20.29 (out of a total score of 56; Table 1). Using the cut-off point of 14 in the questionnaire, 65.2% of caregivers presented with anxiety.

All variables met the assumptions of normality, except for functional capacity.

The descriptive data for coping strategies are presented in Table 2. The strategies most frequently used by the caregivers were acceptance, active coping and the use of emotional support; substance use, behavioral disengagement and humor were the least used.

In terms of the bivariate analysis (Table 3), anxiety was positively associated with the coping strategies of self-distraction (r = 0.16; *p* = 0.031) and denial (r = 0.21; *p* = 0.002), with the sex of the caregiver (r = 0.18; *p* = 0.01) and with subjective burden (r = 0.30; *p* = 0.000), whereas acceptance (r = −0.15; *p* = 0.024) was negatively associated.

An independent regression model was constructed for anxiety with each of the types of coping, controlling for sex, functional capacity (log transformed), cognitive decline and subjective burden (Table 4). In these models, anxiety was negatively associated with planning (partial r = −0.18), acceptance (partial r = −0.22) and humor (partial r = −0.20), and was positively associated with self-distraction (partial r = 0.19), venting (partial r = 0.22), denial (partial r = 0.27 and self-blame (partial r = 0.25). 

Except for the absence of normality for the functional capacity variable, no violations of the assumptions were detected in the regression model (Durbin–Watson statistic between 1.58 and 1.788 and tolerance between 0.791 and 0.968).

## 4. Discussion

The strengths of this study lie in the use of an adequate sample size to provide sufficient statistical power, and in adjusting for potential confounding factors. Moreover, the population sample of our study is similar to that of the survey of the Spanish Institute of Social Services (IMSERSO in Spanish) about care of older persons in Spanish homes in its 2005 edition [28], which was representative at a national level. Therefore, our study sample could be considered representative of the caregivers of dependent older adults in Spain, which supports the external validity of this study.

In this study, the most frequent coping strategies used by caregivers of dependent older adult relatives were acceptance, active coping and the use of emotional support. This is consistent with the Mediterranean informal system of care, in which the predominant family support network in the care of dependent older adult is one of its characteristics [29]. 

Furthermore, in this study, after controlling for possible confounding factors (sex, functional capacity, cognitive decline and subjective burden), coping strategies including planning, acceptance and humor were accompanied by less anxiety, while self-distraction, venting, denial and self-blame were accompanied by more anxiety. 

Planning is considered an active coping strategy or approximation centered on the resolution of problems [30]. Our results are consistent with those of other studies [31,32] that showed that caregivers who use active coping strategies focused on the resolution of problems experience less anxiety and better psychological adaptation. Our data reveal that caregivers who use a planning strategy experience less anxiety, and therefore this may be a protective strategy for anxiety. 

Acceptance and humor have been classified as active emotion-focused coping strategies [30]. Our findings are similar to those found in systematic reviews [15,18] and prior original studies on the caregivers of dependent persons [16,33] that found that anxiety was negatively associated with active emotion-focused coping strategies. Hence, acceptance and humor coping strategies may be protective against anxiety. Our results support the hypothesis that the use of emotion-focused coping strategies are more effective in situations of low control [34], such as caring for a dependent older person. The decrease in anxiety related to the use of acceptance and humor could be due to a decrease in the caregiver’s perception of the situation as being stressful. 

Self-distraction, venting, denial and self-blame have been classified as passive and dysfunctional coping strategies [30]. Our results demonstrate that the use of these types of coping strategies are accompanied by higher levels of anxiety in caregivers. These results are consistent with those found in other studies [17,18,19]. Furthermore, these results show that avoiding the challenges of the caregiving situation could cause anxiety and make the identification of more-effective coping strategies more difficult. Therefore, the use of self-distraction, venting, denial and self-blame coping strategies could be a risk factor for anxiety.

Our results support the hypothesis that interventions based on acceptance [34,35] and problem-solving [36] are needed to decrease anxiety in caregivers of dependent older adult relatives.

This study has some limitations. First, its descriptive cross-sectional design means that no casual conclusions can be made among variables. Second, the use of a convenience sample could affect the external validity; however, the sample is similar to that of a representative national study (as mentioned above) and the number of caregivers who declined participation in the study was low enough to reduce the influence of this limitation.

## 5. Conclusions

Despite the aforementioned limitations, we are able to make the following conclusions: (1) the coping strategies most used by caregivers of dependent older adult relatives are acceptance, active coping and the use of emotional support; (2) planning, acceptance and humor coping strategies are protective factors for anxiety; and (3) self-distraction, venting, denial and self-blame strategies may be risk factors for anxiety. Our results support conducting an initial evaluation of coping strategies used by caregivers, as well as the implementation of interventions based on acceptance and problem-solving to decrease anxiety in the caregivers of dependent older adult relatives. Longitudinal studies are needed to expand evidence on the relationship between coping and anxiety.

## Figures and Tables

**Table 1:** Sample description.

Variables		*n* (%)	M (SD)	95% CI
**Caregiver**				
Age			58.2 (12.8)	56.32; 60.07
Gender				
	Female	177 (89.4%)		84.85; 93.93
	Male	21 (10.6%)		6.06; 15.14
Relationship				
	Spouse	68 (34.3%)		27.47; 41.21
	Offspring	113 (57.1%)		49.92; 64.21
	Other	17 (8.6%)		4.43; 12.74
Common residence		138 (69.7%)		63.04; 76.35
Duration of caregiving in months			35.7 (46.1)	29.04; 42.47
NADLs			5.57 (4.26)	4.95; 6.19
HADLs			2.71 (2.65)	2.32; 3.09
HARS (Range 0–56)			20.29 (10.76)	18.78; 21.80
CSI (Range 0–13)			5.87 (2.83)	5.46; 6.28
**Care Recipient**				
Age			78.1 (8.1)	77.00; 79.38
Gender				
	Male	103 (52%)		44.80; 59.23
	Female	95 (48%)		40.76; 55.19
Main pathology				
	Stroke	48 (24.2%)		18.02; 30.46
	Cancer	49 (24.7%)		18.48; 31.01
	Dementia	18 (9.1%)		4.83; 13.34
	Other	83 (41.9%)		24.12; 37.49
BI (Range 0–90)			18.15 (19.00)	15.39; 20.92
SPMSQ (Range 0–10)			4.86 (3.13)	4.41; 5.32

M: mean; SD: standard deviation; CI: confidence interval; NADLs: number of activities of daily living that were assisted; HADLs: hours per day of activities of daily living that were assisted; HARS: Hamilton Anxiety Rating Scale; CSI: Caregiver Strain Index; BI: Barthel Index; SPMSQ: short portable mental status questionnaire.

**Table 2:** Frequency of coping strategies.

Coping Strategy	Range	M	SD
Active coping	2–8	6.13	1.759
Planning	2–8	5.83	1.724
Instrumental support	2–8	5.63	1.847
emotional support	2–8	6.04	1.923
Positive reframing	2–8	4.25	2.007
Acceptance	2–8	6.22	1.450
Religion	2–8	5.59	2.142
Humor	2–5	2.41	0.831
Self-distraction	2–8	3.98	1.652
Venting	2–8	4.01	1.639
Denial	2–8	3.86	1.847
Behavioral disengagement	2–8	2.41	1.066
Substance use	2–5	2.19	0.624
Self-blame	2–8	2.96	1.349

SD: standard deviation.

**Table 3:** Correlation matrix of the study variables.

Variables	2	3	4	5	6	7	8	9	10	11	12	13	14	15	16	17	18	19
1 HARS	−0.057	−0.129	−0.001	0.014	0.025	−0.154 *	0.045	−0.118	0.160 *	0.118	0.218 **	−0.017	0.069	0.111	0.184 **	−0.041	−0.038	0.301 **
2 Active coping		0.737 **	0.386 **	0.159 *	0.278 **	0.242 **	−0.297**	0.190 **	0.029	0.234 **	0.010	0.042	−0.055	0.184 **	−0.115	0.116	0.042	0.074
3 Planning			0.397 **	0.184 **	0.303 **	0.366 **	−0.046	0.077	0.001	0.108	0.032	0.071	−0.031	0.054	−0.043	0.064	0.131	0.063
4 instrumental support				0.782 **	0.155 *	0.247 **	0.096	−0.019	−0.011	0.222 **	0.004	0.037	−0.123	−0.097	0.020	0.094	−0.011	−0.186 **
5 emotional support					0.102	0.125	0.243**	−0.108	0.000	0.106	−0.020	−0.017	−0.124	−0.023	0.023	0.163 *	−0.046	−0.246 **
6 Positive reframing						0.223 **	−0.060	0.217 **	0.297 **	0.092	−0.257 **	−0.191 **	−0.100	0.097	0.125	−0.017	0.228 **	−0.210 **
7 Acceptance							0.101	0.092	−0.254 **	0.051	−0.218 **	−0.007	−0.160 *	−0.022	0.121	−0.023	0.212 *	−0.068
8 Religion								−0.170 *	0.051	0.054	0.005	0.048	0.070	−0.175 *	0.210 **	0.146 *	−0.208 *	−0.070
9 Humor									0.080	0.353 **	0.015	−0.114	0.179*	0.113	−0.006	0.216 **	−0.029	0.081
10 Self-distraction										0.129	0.032	−0.090	0.102	0.009	0.175 *	−0.121	0.085	0.099
11 Venting											0.274 **	0.069	0.034	0.002	0.172 *	0.192 **	−0.004	0.065
12 Denial												0.159 *	0.116	0.090	0.116	0.121	−0.129	0.221 **
13 Behavioral dis.													0.078	0.095	−0.113	0.122	−0.249 **	0.129
14 Substance use														0.044	−0.131	−0.002	−0.062	0.069
15 Self-blame															−0.204 **	0.086	−0.119	0.085
16 Caregiver sex																−0.080	0.145	0.088
17 BI																	−0.375 **	0.029
18 SPMSQ																		−0.063
19 CSI																		

* The correlation is significant at level 0.05 (bilateral). ** The correlation is significant at level 0.01 (bilateral). HARS: Hamilton Anxiety Rating Scale; BI: Barthel Index; SPMSQ: short portable mental status questionnaire; CSI: Caregiver Strain Index.

**Table 4:** Regression models of the different types of coping and anxiety, controlling by sex, functional capacity, cognitive deterioration and subjective burden.

Independent Variable in Each Model	Partial r	Partial r^2^	*p*-Value	r^2^ (1)
Active coping	−0.007	0.000	0.941	0.147
Planning	−0.182	0.033	**0.042**	0.175
Instrumental support	0.005	0.000	0.952	0.147
Emotional support	0.054	0.003	0.550	0.149
Positive reframing	0.049	0.002	0.584	0.149
Acceptance	−0.221	0.049	**0.013**	0.189
Religion	0.015	0.000	0.866	0.147
Humor	−0.197	0.039	**0.027**	0.180
Self-distraction	0.185	0.034	**0.038**	0.176
Venting	0.216	0.047	**0.015**	0.187
Denial	0.273	0.074	**0.002**	0.211
Behavioural disengagement	0.007	0.000	0.937	0.147
Substance use	0.057	0.003	0.525	0.150
Self-blame	0.247	0.061	**0.005**	0.199

(1) r^2^ of the whole model (independent variable plus control variables); *p* < 0.05 are in bold text.
